# Aha1 Exhibits Distinctive Dynamics Behavior and Chaperone-Like Activity

**DOI:** 10.3390/molecules26071943

**Published:** 2021-03-30

**Authors:** Huifang Hu, Qing Wang, Jingwen Du, Zhijun Liu, Yiluan Ding, Hongjuan Xue, Chen Zhou, Linyin Feng, Naixia Zhang

**Affiliations:** 1 Analytical Research Center for Organic and Biological Molecules, Shanghai Institute of Materia Medica, Chinese Academy of Sciences, 555 Zu Chong Zhi Road, Shanghai 201203, China; 201628012342108@simm.ac.cn (H.H.); s18-dujingwen@simm.ac.cn (J.D.); ylding@simm.ac.cn (Y.D.); 2 University of the Chinese Academy of Sciences, 19A Yuquan Road, Beijing 100049, China; 201628012342065@simm.ac.cn; 3 CAS Key Laboratory of Receptor Research, Shanghai Institute of Materia Medica, Chinese Academy of Sciences, Shanghai 201203, China; 4 National Facility for Protein Science in Shanghai, ZhangJiang Lab, Shanghai Advanced Research Institute, Chinese Academy of Sciences, Shanghai 201210, China; liuzhijun@sari.ac.cn (Z.L.); xuehongjuan@sari.ac.cn (H.X.)

**Keywords:** Aha1, NMR, dynamics, chaperone-like activity, α-synuclein

## Abstract

Aha1 is the only co-chaperone known to strongly stimulate the ATPase activity of Hsp90. Meanwhile, besides the well-studied co-chaperone function, human Aha1 has also been demonstrated to exhibit chaperoning activity against stress-denatured proteins. To provide structural insights for a better understanding of Aha1’s co-chaperone and chaperone-like activities, nuclear magnetic resonance (NMR) techniques were used to reveal the unique structure and internal dynamics features of full-length human Aha1. We then found that, in solution, both the two domains of Aha1 presented distinctive thermal stabilities and dynamics behaviors defined by their primary sequences and three-dimensional structures. The low thermal stability (melting temperature of Aha1^28–162^: 54.45 °C) and the internal dynamics featured with slow motions on the µs-ms time scale were detected for Aha1’s N-terminal domain (Aha1N). The aforementioned experimental results suggest that Aha1N is in an energy-unfavorable state, which would therefore thermostatically favor the interaction of Aha1N with its partner proteins such as Hsp90’s middle domain. Differently from Aha1N, Aha1C (Aha1’s C-terminal domain) exhibited enhanced thermal stability (melting temperature of Aha1^204–335^: 72.41 °C) and the internal dynamics featured with intermediate motions on the ps-ns time scale. Aha1C’s thermal and structural stabilities make it competent for the stabilization of the exposed hydrophobic groove of dimerized Hsp90’s N-terminal domain. Of note, according to the NMR data and the thermal shift results, although the very N-terminal region (M1-W27) and the C-terminal relaxin-like factor (RLF) motif showed no tight contacts with the remaining parts of human Aha1, they were identified to play important roles in the recognition of intrinsically disordered pathological α-synuclein.

## 1. Introduction

Proteins are essential molecules for all living systems, and the majority of proteins depend on a well-defined shape (three-dimensional structure) to obtain their functionalities [1,2,3]. Molecular chaperones are key components of a protein quality control system and a protein class that assist client proteins in forming the correct three-dimensional structures and prevent protein misfolding and/or aggregation [3,4,5,6,7,8]. According to the functional similarities, molecular chaperones are classified as ATPase chaperones, holdase chaperones, chaperonins, translocases, insertases, etc. [9]. The well-known Hsp90 belongs to the ATPase chaperone group, and the hydrolysis of ATP drives conformational changes of Hsp90 associated with its different function stages: Hsp90 in the apo state exists in a “V”-shaped conformation dimerized via its C-terminal domain; ATP binding triggers Hsp90 switching to a closed catalytically active state, and the N-terminal domain of each Hsp90 protomer binds together; with the hydrolysis of ATP and the releasing of ADP, Hsp90 returns to its apo state [10,11,12,13,14,15]. Besides ATP and ADP molecules, quite a few co-chaperone proteins are also involved in regulating the functional display of Hsp90. Among the identified co-chaperones, Aha1 (Activator of Hsp90 ATPase protein 1) is the only one known to strongly accelerate the ATPase cycle of Hsp90 [16,17,18,19,20,21,22].

Aha1 is composed of two structural domains (the N-terminal domain and the C-terminal domain), which are connected by a long linker region [17,22,23,24,25,26,27]. It has been demonstrated that Aha1 modulates Hsp90’s activity in an asymmetric way. One Aha1 molecule per Hsp90 dimer is sufficient for a full stimulation of Hsp90’s ATPase activity [22]. The occurrence of the stimulation is driven by two main interactions between Aha1 and Hsp90: at first, the N-terminal domain of Aha1 interacts with the middle domain of Hsp90 and induces the conformational rearrangements of Hsp90 that favor the N terminal domain-dimerized state of the chaperone; then, the exposed hydrophobic binding groove formed by both N-terminal domains of Hsp90 protomers is stabilized via its interaction with the C-terminal domain of Aha1 [17,18,21,22,24]. Of note, it is also suggested that Aha1 might regulate the dwell time of the client protein on Hsp90 [21,27]. The down-regulation/knocking-out of Aha1 presented a rescue effect on misfolded cystic fibrosis transmembrane conductance regulator (CFTR) in mammals [27,28,29] and an activation effect on glucocorticoid receptor (GR) in yeast [30].

**Figure 1:**
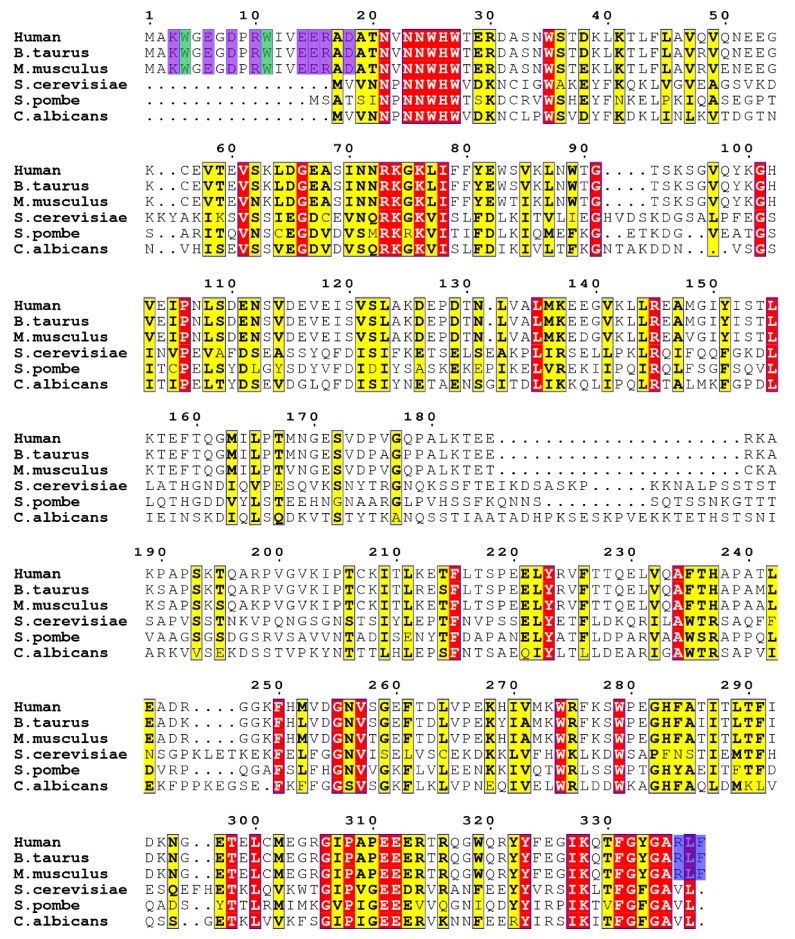
Sequence alignment of representative Aha1 homologs from different species. The conserved residues are marked in red. The charged residues and the tryptophan residues in the very N-terminal region of Aha1 are highlighted in purple and green, respectively. The C-terminal RLF motif of Aha1 from higher eukaryotes is highlighted in blue. The sequence alignment result was generated by using CLUSTALW [32] and ESPript 3.0 [33].

Unlike the Hsp90 family, which presents a highly conserved sequence similarity across the species, Aha-type co-chaperones from different species share a low sequence identity (Figure 1). For example, although the yeast Aha1 (Activator of Hsp90 ATPase protein 1) and the human Aha1 (Activator of 90 kDa heat shock protein ATPase homolog 1, Ahsa1) stimulate Hsp90’s activity in a similar manner, their sequence identity is as low as 23% [24,31] (Figure 1). In particular, an additional fragment featured with tryptophan and charged residues shows in the very N-terminal region of human Ahsa1 but absent from yeast Aha1 (Figure 1). This fragment has been identified to allow Ahsa1 to act as an autonomous chaperone, which prevents the stress-denatured proteins from aggregation [25]. Since human Aha1 possesses a unique primary sequence, a distinctive high-order structure is expected. In this manuscript, NMR techniques were used to solve the solution structures and to characterize the dynamics behaviors of human Aha1. According to the obtained NMR and thermal shift assay data, although both the N-terminal domain and the C-terminal domain of human Aha1 form globule-like structures in solution, their thermal stabilities are extremely different from each other. Consistent with the low thermal stability of Aha1’s N-terminal domain, it exhibited a distinctive dynamic behavior featured with slow motion on a time scale of microseconds to milliseconds (μs to ms) in solution. In addition, we found that the existence of the additional fragment featured with tryptophan and charged residues in the very N-terminal region of Aha1 presented no significant effect on the thermal stability and the core structure of Aha1. However, the presence of Aha1’s very N-terminal fragment and/or the existence of Aha1’s C-terminal RLF motif were identified to play important roles in the interaction between Aha1 and α-synuclein (one of the well-known intrinsically disordered proteins). Moreover, both the α-synuclein fibril formation assay and ThT experiment results indicated that the binding of Aha1 to α-synuclein would inhibit the aggregation of the IDP (intrinsically disordered protein). The possible working mechanism of Aha1’s chaperone-like activity was discussed in the manuscript.

## 2. Results

### 2.1. Solution Structure Determination of Human Aha1’s N-Terminal Domain

**Figure 2:**
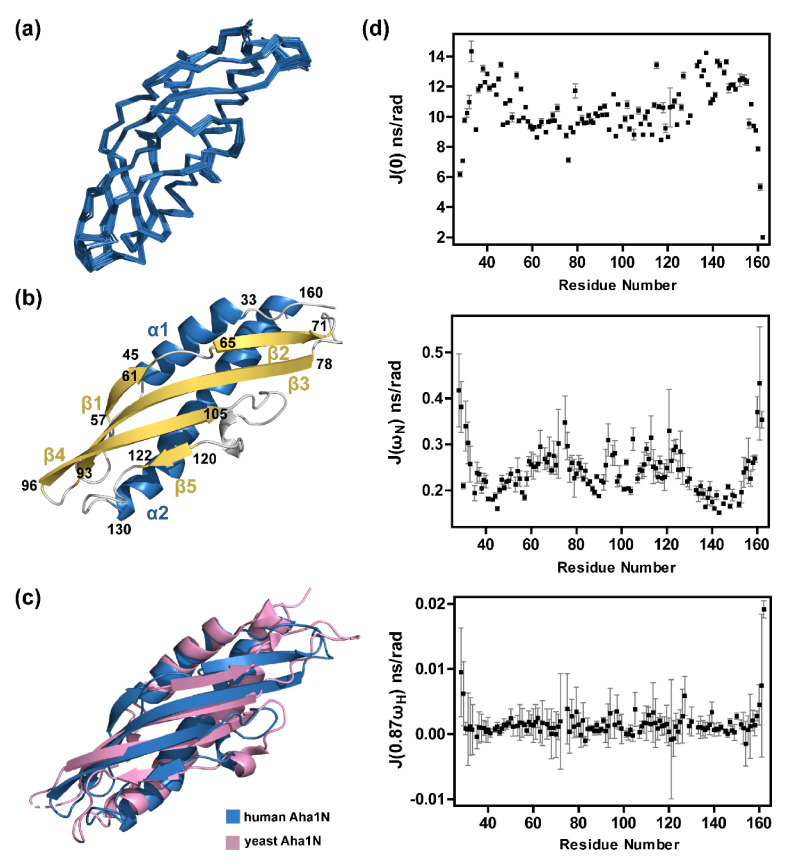
Solution structure and dynamics behavior of human Aha1’s N-terminal domain. (**a**) Structural ensemble of the 20 best stuctures of human Aha1’s N-terminal domain (human Aha1N, PDB code: 7DMD). (**b**) Ribbon representation of the lowest-energy structure of human Aha1’s N-terminal domain. (**c**) Superposition of the structures for human Aha1N (blue, PDB code: 7DMD) and yeast Aha1N (pink, PDB code: 1USU). (**d**) Reduced spectral density functions of human Aha1’s N-terminal domain in its free state.

To solve the solution structure of Aha1’s N-terminal domain, we produced Aha1^1−162^ protein sample and ran the stability test (data not shown). We then found that the existence of the very N-terminal region of Aha1’s N-terminal domain would decrease the stability of Aha1^1−162^ and lead to protein degradation. Therefore, Aha1^28−162^ instead of Aha1^1−162^ was subjected to NMR studies. Multiple NMR techniques were jointly applied to determine the solution structure ensemble of Aha1^28−162^ (Table 1, Figure 2a–c). The studies revealed that human Aha1^28−162^ in solution state adopts an elongated cylindrical fold similar to the reported crystal structure of yeast Aha1’s N-terminal domain (in complex with yeast Hsp90’s middle domain) (Figure 2a–c). In particular, one anti-parallel β-sheet comprising two contiguous β-strand (E57-V61 and D65-N71) and three consecutive β-strands (I78-S93, G96-I105, and S120-S122) are formed, and this sheet packs against two α-helices spanning S33-L45 and T130-T160.

After the solution structure determination of human Aha1’s N-terminal domain, ^15^N relaxation experiments to measure amide longitudinal (R_1_) and transverse (R_2_) relaxation rates, and amide heteronuclear NOE enhancement (XNOE) experiments to detect XNOE values, were recorded by using ^15^N, 50% ^2^H-double-labeled Aha1^28−162^ (Supplemental Figure S1). The reduced spectral density mapping (SDM) method [34,35,36,37,38] was then utilized to interpret the relaxation data. The spectral densities J(0), J(ω_N_) and J(0.87ω_H_) were calculated to describe the internal motions of Aha1^28−162^ at low, intermediate and high frequencies, respectively (Figure 2d). In particular, the transverse relaxation (R_2_) is dramatically sensitive to slow motions at μs-ms scale (such as molecular tumbling and conformational/chemical exchange), which are described by the spectral density of J(0) [38]. The amide longitudinal relaxation (R_1_) is sensitive to both the intermediate and the high-frequency motions at ps-ns scale (described by J(ω_N_) and J(0.87ω_H_), respectively). Increased intermediate and high-frequency motions enhance R_1_ relaxation. However, R_1_ is mostly affected by the changes in J(ω_N_) [38]. The XNOE is also sensitive to both J(ω_N_) and J(0.87ω_H_), and J(ω_N_) decreasing and J(0.87ω_H_) increasing will lead to XNOE reduction [38]. According to the determined J(0.87ω_H_) for amino acid residues in Aha1^28−162^ (Figure 2d), only a limited variation in J(0.87ω_H_) values was observed for almost all of the residues, except those located at the two ends of Aha1^28−162^. These dynamics data are consistent with the compacted structural arrangements of Aha1^28−162^, and only the unstructured N-terminal and C-terminal regions of the protein exhibited high flexibilities featured with high-frequency motions in solution (Figure 2). Meanwhile, it is worth noting that the J(0) values for most of the residues in two α-helices (S33-L45 and T130-T160) of Aha1^28−162^ are larger than the J(0) values for those residues in the remaining well-structured regions of the protein (Figure 2d). These data indicate that the two α-helices of Aha1^28−162^ underwent more slow motions in solution and would therefore be less stable.

### 2.2. Aha1’s N-Terminal Domain Presents Unique Dynamics Behavior and Structural Features Different from Aha1’s C-Terminal Domain

**Figure 3:**
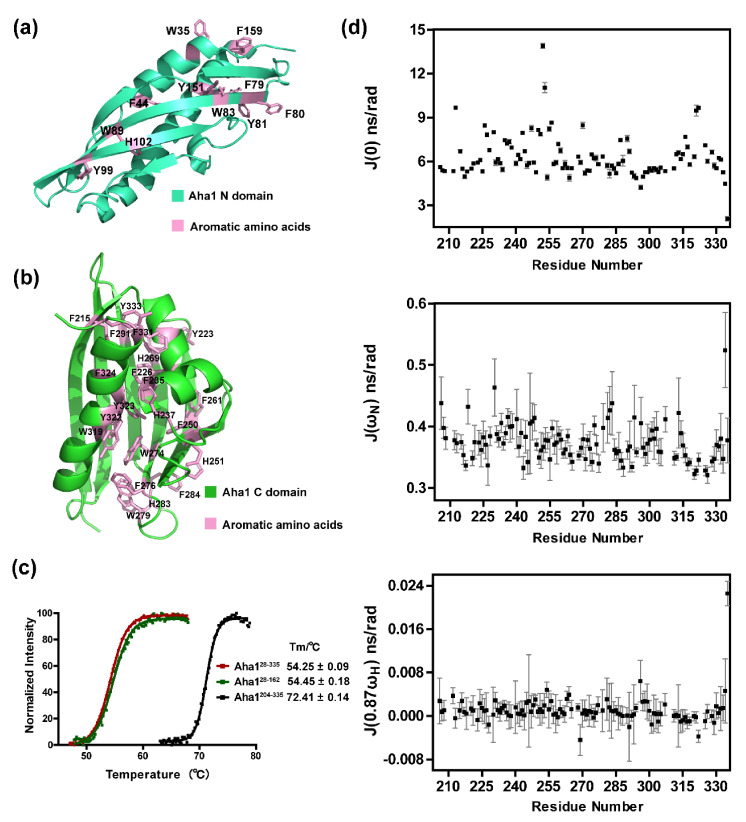
Aha1’s C-terminal domain presents unique dynamics behavior and structural features different from Aha1’s N-terminal domain. (**a**) Ribbon representation of the solution structure of human Aha1’s N-terminal domain (Aha1^28−162^). Aromatic residues (W35, F44, F79, F80, Y81, W83, W89, Y99, H102, Y151 and F159) are highlighted in pink. (**b**) Ribbon representation of the solution structure of human Aha1’s C-terminal domain (Aha1^204−335^, PDB code: 1 × 53). Aromatic residues (F215, Y223, F226, F235, H237, F250, H251, F261, H269, W274, F276, W279, H283, F284, F291, W319, Y322, Y323, F324, F331 and Y333) are highlighted in pink. (**c**) The Tm values of Aha1^28−162^, Aha1^204−335^ and Aha1^28−335^ were determined. The measured melting temperatures for Aha1^204−335^, Aha1^28−162^ and Aha1^28−335^ are 72.41 ± 0.14 °C, 54.45 ± 0.18 °C and 54.25 ± 0.09 °C, respectively. (**d**) Reduced spectral density functions of human Aha1’s C-terminal domain (Aha1^204−335^) in its free state.

The solution structure of human Aha1’s C-terminal domain (Aha1C) was solved by Tochio et al. of RIKEN Structural Genomics/Proteomics Initiative (RSGI) in 2005 (Aha1^204−335^, PDB code: 1 × 53). Compared with human Aha1’s N-terminal domain (Aha1N), Aha1C forms a more globule-like fold in solution (Figure 3a,b). The three-dimensional configuration of Aha1C is stabilized by Van der Waals interactions mainly mediated by the 21 aromatic residues, namely F215, Y223, F226, F235, H237, F250, H251, F261, H269, W274, F276, W279, H283, F284, F291, W319, Y322, Y323, F324, F331 and Y333 (Figure 3b). It is worth noting that although the sizes of the well-structured core regions for Aha1N (Aha1^28−162^) and Aha1C (Aha1^204−335^) are similar to each other, Aha1^28−162^ contains a much less number of aromatic residues in its primary sequence (Figure 3a: W35, F44, F79, F80, Y81, W83, W89, Y99, H102, Y151 and F159). At least partially due to the stronger Van der Waals interactions mediated by aromatic residues, Aha1C (Aha1^204−335^) exhibited a higher thermal stability than Aha1N (Aha1^28−162^) (Figure 3c). The measured melting temperatures for Aha1C, Aha1N and Aha1^28−335^ are 72.41 ± 0.14 °C, 54.45 ± 0.18 °C and 54.25 ± 0.09 °C, respectively. The unique structural features and also the structure-determined thermal stability of Aha1C was further supported by the ^15^N relaxation data (Figure 3d, Supplemental Figure S2). The average J(0) value for all residues of Aha1^204−335^ is 6.29 ± 0.13 ns/rad, which is significantly smaller than the corresponding mean J(0) value of 10.49 ± 0.16 ns/rad for all residues of Aha1^28−162^. The smaller mean J(0) value indicates that less slow motions such as slow conformational/chemical exchanges occur in Aha1^204−335^.

**Figure 4:**
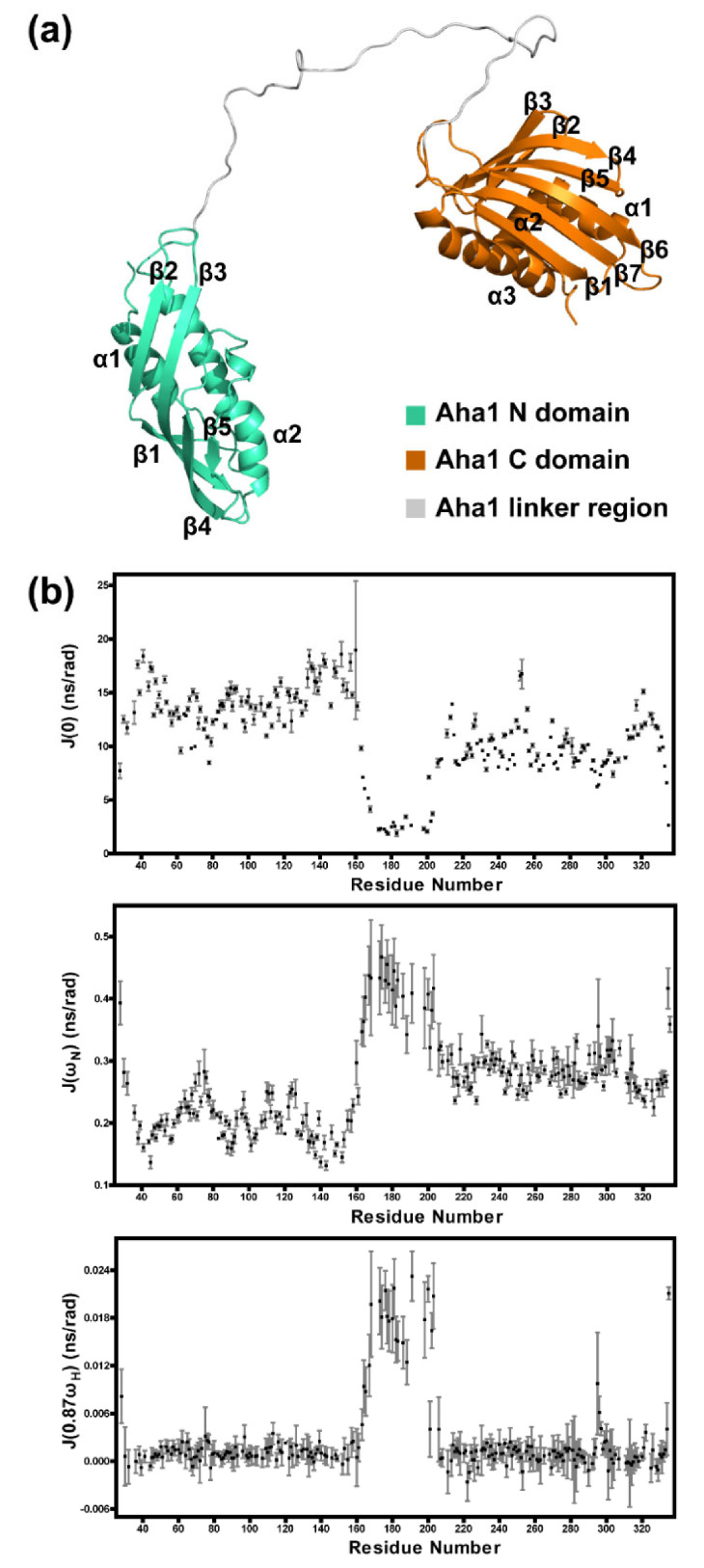
Aha1’s N-terminal domain and C-terminal domain are linked through a long unstructured loop, which confers a low restriction to the relative positioning of these two domains in solution. (**a**) Ribbon representation of the solution structure of human Aha1^28−335^. The N-terminal domain and the C-terminal domain of Aha1 are colored in cyan and golden orange, respectively. (**b**) Reduced spectral density functions of human Aha1^28−335^.

After the structure and dynamics characterizations of the N-terminal domain and the C-terminal domain of human Aha1, NMR techniques were applied to determine the solution structure of Aha1^28−335^. Since the fingerprint spectra (^1^H-^15^N-HSQC) of Aha1^28−162^ and Aha1^204−335^ superimposed well with the ^1^H-^15^N-HSQC of Aha1^28−335^ (Supplemental Figure S3), the calculation constraints for most of the residues (except those residues in the fragments of L155-G162 and I204-T206) in Aha1^28−162^ and Aha1^204−335^ (PDB code: 1X53) were directly transferred and applied in the structure calculations of Aha1^28−335^. Besides, NOE-derived distance restraints and dihedral angle restraints for the residues in fragment L155-T206 were generated by the analysis of ^15^N-NOESY-HSQC spectrum and the chemical shifts of C, CA, CB, N and HN of the corresponding residues in Aha1^28−335^, respectively. The aforementioned restraints and the RDC restraints extracted from the ^15^N-IPAP-HSQC spectra of Aha1^28−335^ were jointly applied in the structure calculations of Aha1^28−335^ (Table 1). The solved solution structure ensemble of Aha1^28−335^ indicates that human Aha1’s N-terminal domain and C-terminal domain are linked through a long unstructured loop (Figure 4a, Supplemental Figure S4), and there are no tight contacts between the two domains.

In the following studies, ^1^H-^15^N-HSQC titrations and ^15^N relaxation experiments were conducted to verify the achieved solution structure ensemble of Aha1^28−335^. ^1^H-^15^N-HSQC spectra were recorded on ^15^N-labeled Aha1^28−162^ without or with the presence of equal molar of unlabeled Aha1^204−335^ and ^15^N-labeled Aha1^204−335^ without or with the presence of equal molar of unlabeled Aha1^28−162^. Since no significant chemical shift perturbations were detected for both ^15^N-labeled Aha1^28−162^ and ^15^N-labeled Aha1^204−335^ upon the presence of their unlabeled partner proteins (Supplemental Figure S5), we then conclude that the two domains of human Aha1 do not interact with each other in solution. These data are consistent with the obtained solution structure ensemble of Aha1^28−335^, in which a quite flexible spatial arrangement for Aha1’s N-terminal and C-terminal domains was revealed (Supplemental Figure S4). Meanwhile, Aha1^28−335’^s solution structure ensemble was also verified by the ^15^N relaxation data (Figure 4b, Supplemental Figure S6). A long-loop region spanning M163-T206 featured with both the intermediate and the high-frequency motions at ps-ns time scale (described by J(ω_N_) and J(0.87ω_H_), respectively) was clearly identified (Figure 4b). Additionally, compared with the observations in the dynamics data of Aha1’s N-terminal domain (Aha1N, Aha1^28−162^) and Aha1’s C-terminal domain (Aha1C, Aha1^204−335^) (Figure 2d and Figure 3d), the same dynamic features for these two structural domains were revealed by carrying out the relaxation experiments on Aha1^28−335^ (Figure 4b). High-intensity slow motions at μs-ms time scale were observed for Aha1N, and more internal motions at ps-ns time scale (J(ω_N_)) occurred in Aha1C (Figure 4b). Therefore, the correctness of Aha1^28−335’^s global fold determined by NMR techniques, in which two independent domains are linked together by a long unstructured loop region, was further confirmed by the dynamics data.

### 2.3. The Very N-Terminal Region and the C-Terminal RLF Motif of Aha1 Show No Significant Effects on the Global Fold of the Protein

**Figure 5:**
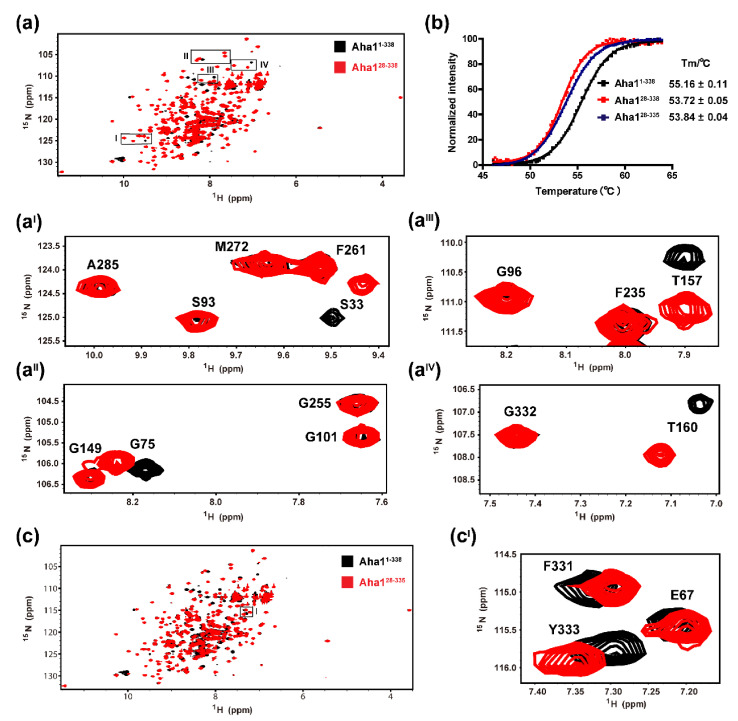
The very N-terminal region and the C-terminal RLF motif of Aha1 present no significant effect on the global fold of the protein. (**a**,**a^I^**,**a^II^**,**a^III^**,**a^IV^**) Superposition of ^1^H-^15^N-HSQC spectra recorded on ^15^N-labeled Aha1^1−338^ (colored in black) and ^15^N-labeled Aha1^28−338^ (colored in red). Selected ^1^H-^15^N-HSQC spectra regions are expanded to view representative residues that underwent resonance shifting upon the absence of Aha1’s very N-terminal fragment (M1-W27). (**b**) The measured Tm values for Aha1^1−338^, Aha1^28−338^ and Aha1^28−335^ were 55.16 ± 0.11 °C, 53.72 ± 0.05 °C and 53.84 ± 0.04 °C, respectively. (**c**,**c^I^**) Superposition of ^1^H-^15^N-HSQC spectra recorded on ^15^N-labeled Aha1^1−338^ (colored in black) and ^15^N-labeled Aha1^28−335^ (colored in red). Selected ^1^H-^15^N-HSQC spectra regions are expanded to view representative residues that undergo resonance shifting upon the absence of Aha1’s C-terminal RLF motif.

According to the available bioinformatics data and the published literature, the very N-terminal region of human Aha1 (M1-V22) is featured with tryptophan and charged residues (Figure 1), and the existence of this special fragment (M1-V22) allows human Aha1 to act as an autonomous chaperone and prevent the stress-denatured proteins from aggregation [25]. To test if the very N-terminal region forms tight contacts with the remaining part of human Aha1, ^1^H-^15^N-HSQC experiments and thermal shift assay were carried out. Since most of the signals in the fingerprint spectrum (^1^H-^15^N-HSQC) of Aha1^28−338^ superimposed well with those corresponding peaks in the ^1^H-^15^N-HSQC spectrum of Aha1^1−338^ (Figure 5a), we then conclude that Aha1’s very N-terminal fragment do not form tight contacts with the remaining part of human Aha1 in solution. Upon the presence of the fragment spanning M1-W27, no significant global fold change occurred in the region spanning T28-F338 of Aha1. A detailed ^1^H-^15^N-HSQC data analysis revealed that with the attachment of fragment M1-W27, only those residues located in either the linked region or the spatially close regions to M1-W27 (T28-S33, S69-L77 and L155-T167) underwent significant chemical shift perturbations (Figure 5a, Supplementary Figure S7). Moreover, inconsistently with the aforementioned conclusion that the very N-terminal region of Aha1 showed no significant effect on the global fold of the protein, only a very limited thermal stability difference between Aha1^1−338^ and Aha1^28−338^ was detected (Figure 5b). The determined thermal shift value for Aha1^1−338^ vs. Aha1^28−338^ is 1.44 °C.

In the following study, ^1^H-^15^N-HSQC experiment and thermal shift assay were conducted to reveal if the presence of Aha1’s three C-terminal residues (R^336^L^337^F^338^) would induce global structure changes of Aha1. Like that indicated by the superimposed ^1^H-^15^N-HSQC spectra of Aha1^1−338^ and Aha1^28−335^ (Figure 5c, Supplementary Figure S8), most of the signals in the fingerprint spectrum (^1^H-^15^N-HSQC) of Aha1^28−335^ superimposed well with those corresponding peaks in the ^1^H-^15^N-HSQC spectrum of Aha1^1−338^ (Figure 5c). These data suggest that the presence of Aha1’s three C-terminal residues (R^336^L^337^F^338^) would not induce significant global structure changes of Aha1. A detailed ^1^H-^15^N-HSQC data analysis revealed that with the attachment of the three C-terminal residues, most of the residues locating in the linked region to R^336^L^337^F^338^ (G326-A335) underwent significant chemical shift changes (Supplemental Figure S7). Additionally, the HSQC signal of residue L216, which is located in the loop region (F215-S218) spatially close to Aha1’s C-terminus, was also significantly perturbed (Supplementary Figure S7). Moreover, inconsistently with the aforementioned conclusion that the C-terminal RLF motif of Aha1 showed no significant effect on the global fold of the protein, only a very limited thermal stability difference for Aha1^28−338^ vs. Aha1^28−335^ was detected (Figure 5b). The determined thermal shift value for Aha1^28−338^ vs. Aha1^28−335^ is 0.12 °C.

### 2.4. Both the N-Terminal Fragment M1-W27 and the C-Terminal RLF Motif of Aha1 Contribute to the Recognition of α-Synuclein

**Figure 6:**
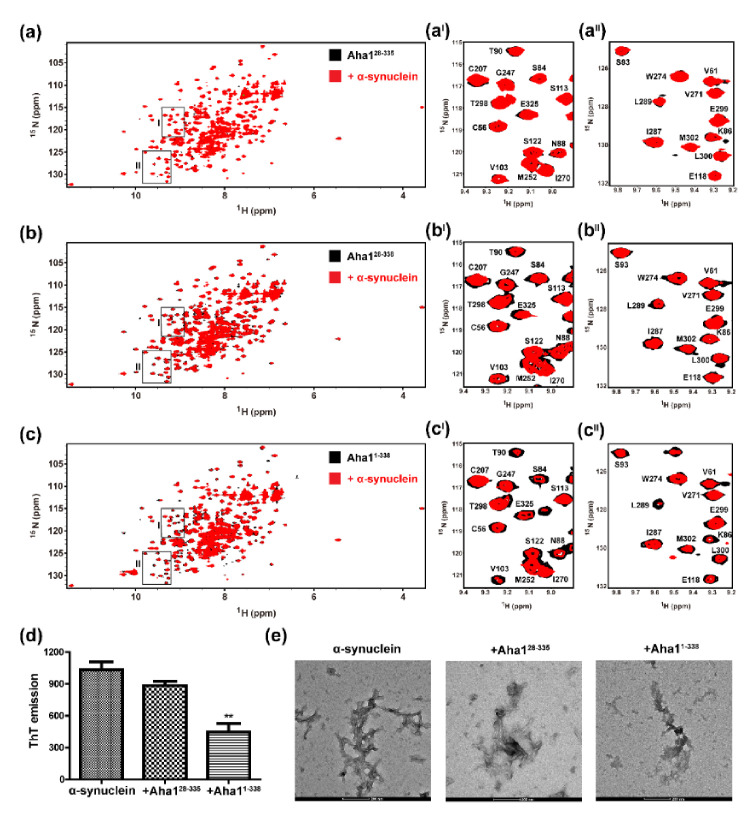
Both the N-terminal fragment M1-W27 and the C-terminal RLF motif of Aha1 contribute to the recognition of α-synuclein. (**a**,**a^I^**,**a^II^**) Superposition of ^1^H-^15^N-HSQC spectra recorded on ^15^N-labeled Aha1^28−335^ without (colored in black) or with (colored in red) the addition of 10-fold molar excess of unlabeled α-synuclein. (**b**,**b^I^**,**b^II^**) Superposition of ^1^H-^15^N-HSQC spectra recorded on ^15^N-labeled Aha1^28−338^ without (colored in black) or with (colored in red) the addition of 10-fold molar excess of unlabeled α-synuclein. Selected ^1^H-^15^N-HSQC spectra regions are expanded to view representative residues that undergo resonance attenuations upon the presence of α-synuclein. (**c**,**c^I^**,**c^II^**) Superposition of ^1^H-^15^N-HSQC spectra recorded on ^15^N-labeled Aha1^1−338^ without (colored in black) or with (colored in red) the addition of 10-fold molar excess of unlabeled α-synuclein. Selected ^1^H-^15^N-HSQC spectra regions are expanded to view representative residues that undergo resonance attenuations upon the presence of α-synuclein. (**d**) The ThT data indicates that full-length Aha1 exhibits an inhibition effect on the aggregation process of α-synuclein. Decreased ThT emission was observed when Aha1^1−338^ but not Aha1^28−335^ was premixed with α-synuclein before the further incubation (*n* = 3; ** *p* < 0.05). (**e**) Inhibitory effect of full-length Aha1 on α-synuclein aggregation. Transmission electron microscopy (120 kV) of α-synuclein fibril (14 μM) incubated with 1.4 μM Aha1 for three days (scale bar: 200 nm).

According to the published literature [25], it seems that the presence of the N-terminal fragment M1-V22 confers upon human Aha1 holdase-like chaperone function. In this study, to test if the N-terminal fragment M1-W27 of Aha1 plays a role in the recognition of disordered proteins, intrinsically disordered α-synuclein was selected as a model protein. ^1^H-^15^N-HSQC spectra using ^15^N-labeled Aha1^1−338^, ^15^N-labeled Aha1^28−338^ and ^15^N-labeled Aha1^28−335^ without or with the presence of 10-fold molar excess of unlabeled α-synuclein were recorded (Figure 6). According to the acquired ^1^H-^15^N-HSQC titration data, both the N-terminal fragment M1-W27 and the C-terminal RLF (R^336^L^337^F^338^) motif of Aha1 contributed to the recognition of α-synuclein. Without the presence of M1-W27 fragment and RLF (R^336^L^337^F^338^) motif, no significant chemical shift perturbations were observed for the NMR resonances of the residues in Aha1^28−335^ upon the addition of α-synuclein (Figure 6a). Meanwhile, global signal attenuations were detected for Aha1^28−338^ and Aha1^1−338^ upon the presence of α-synuclein (Figure 6b,c). Besides, compared with those signal changes detected for Aha1^28−338^:α-synuclein system, a greater extent of signal attenuations was observed for the Aha1^1−338^:α-synuclein system (Figure 6b,c).

After the confirmation of the interaction between human Aha1 and α-synuclein, ThT assay and transmission electron microscopy (TEM) were applied to test the impact of Aha1 on the aggregation process of α-synuclein. Both the ThT data and the TEM results demonstrated that Aha1 presented an inhibition effect on α-synuclein aggregation (Figure 6d,e). Compared to the ThT emission value for the incubation sample of α-synuclein alone, decreased ThT emission value was detected for the incubation sample of α-synuclein mixed with full-length Aha1 (Figure 6d). Besides, inconsistently with the ThT data, α-synuclein manifested sparse, irregular dots in the presence of Aha1, in contrast to the fibrils that formed with α-synuclein alone (Figure 6e).

## 3. Discussion

Molecular chaperones such as Hsp70 and Hsp90 are key components of the protein quality control system. By working jointly with multiple co-chaperones and endogenous small molecules (ATP/ADP), the chaperones assist in the proper folding of client proteins [11,12,13,14,15,18,19,21,26,39,40]. As a well-recognized chaperone protein, the structure and function of Hsp90 have been extensively studied. Besides, more than 20 co-chaperones have been identified to modulate the functional display of human Hsp90 [16,18,20,21,22,26,30,39,41,42,43,44,45,46,47,48]. Among the cohort of Hsp90 co-chaperone proteins, Aha1 is the only co-chaperone known to strongly accelerate the working cycle of Hsp90 [16,17,18,19,20,21,22]. However, to date, there is no three-dimensional structure for which full-length mammalian Aha1 has been determined. Therefore, in this manuscript, NMR techniques were subjected to reveal human Aha1’s solution structure and internal dynamics features.

Human Aha1 contains two well-folded domains: Aha1 N-terminal domain (Aha1N, Aha1^28−162^) and Aha1 C-terminal domain (Aha1C, Aha1^204−335^). According to our NMR data, Aha1N and Aha1C present structure and dynamics characteristics significantly different from each other. Aha1N forms an elongated cylindrical fold (Figure 2), and Aha1C adopts a more globule-like fold that is stabilized by Van der Waals interactions mediated by a striking number of aromatic residues (Figure 3). According to the published literature, the interactions between aromatic residues play a fundamental role in protein stabilization [49,50]. Therefore, the existence of 21 aromatic residues (phenylalanine, histidine, tyrosine and tryptophan) and the π-π clusters formed by them in Aha1C are expected to significantly improve the thermal stability of the protein domain (Figure 3). Inconsistently with the expectation, Aha1N (melting temperature, 54.45 ± 0.18 °C) and Aha1C (melting temperature, 72.41 ± 0.14 °C) fall into the mesostable protein group and thermostable protein group, respectively [50,51]. Besides the recognized structural features associated with the enhanced thermal stability of Aha1C over Aha1N, distinctively different dynamics behaviors of these two domains were also observed. Generally, a higher protein thermal stability indicates an enhanced global conformational rigidity of the protein [50,51,52]. Here, in the case of Aha1C versus Aha1N, consistently with the thermal stability difference between these two domains, the dynamics behavior of Aha1N is featured with slow motions on a time scale of microseconds to milliseconds (μs to ms) (Figure 2, Figure 3 and Figure 4), which strongly indicate structural instability coupled to slow conformational/chemical exchanges. However, it is worth noting that although Aha1C showed restrained slow motions in solution (Figure 4), it underwent much larger intermediate frequency motions at ps-ns scale than Aha1N (Figure 4). For small-sized proteins, increased intermediate frequency motions usually suggest decreased flexibility featured with high-frequency motions [53,54]. However, for Aha1C and Aha1N, which share a similar size falling into the medium protein size range defined by NMR study, high-frequency internal motions with comparable intensities were observed for them (Figure 4). These data suggest that there is no significant difference between the local flexibilities (high-frequency internal motions) of Aha1C and Aha1N. Overall, according to the detected dynamics data and thermal stability results, it seems that the low-intensity slow motions (μs to ms time scale) of Aha1C played a dominant role in improving the thermal stability of the protein domain. Meanwhile, as has been known, when serving as a co-chaperone to Hsp90, Aha1C has been demonstrated to interact with and consequently stabilize the large hydrophobic surface area within the Hsp90’s N-terminal domain (Hsp90N) that is exposed during its dimerization [22]. Aha1C’s unique structural features (high thermal stability and large intermediate frequency motions) might facilitate its interaction with the unstable hydrophobic surface formed by dimerized Hsp90’s N-terminal domain. In fact, not only the C-terminal domain of Aha1 presents the distinctive structure and dynamics features adapted to its co-chaperoning role, the structural arrangement and dynamics behavior of Aha1^28−335^ were also demonstrated to be supportive of the interaction between the co-chaperone and Hsp90. As shown in Figure 4, Aha1N and Aha1C are connected by a long flexible linker. This unique structure arrangement confers a low restriction to the relative positioning of these two domains in solution (Supplemental Figure S4). A jellyfish-like shape was formed when the human Aha1’s N-terminal domain served as an anchor in the superposition of Aha1^28−335’^s solution structures (Supplemental Figure S4). Therefore, after the initial binding of Aha1N to Hsp90’s middle domain [18,21,22,26], the high flexibility of the full-length Aha1’s structure might reduce the energy barrier and therefore favor the recognition and stabilization of the dimerized Hsp90’s N-terminal domains by Aha1C.

**Figure 7:**
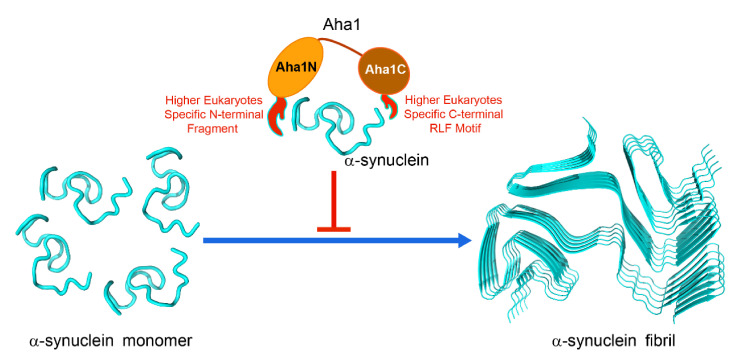
Human Aha1 acts as a holdase-like chaperone and inhibits the aggregation process of α-synuclein in vitro. Part of the presentation was generated by using the reported structure of α-synuclein (PDB code: 6XYO [55]) and the Open-Source PyMOL 1.8.

Beyond the co-chaperone function to Hsp90, Aha1 has also been reported to act as an autonomous chaperone and prevent the stress-denatured proteins from aggregation [25]. Moreover, the chaperone-like function of Aha1 is dependent on the presence of its N-terminal fragment spanning M1-V22 [25]. According to the amino acid compositions of Aha1’s N-terminal region, which are dominated by charged residues and hydrophobic tryptophan residues (Figure 1), we then hypothesized that the involvement of M1-V22 in the interactions between Aha1 and the denatured proteins are potentially triggered by the non-specific electrostatic interactions which are followed by hydrophobic contacts driven by tryptophan residues. Therefore, the representative intrinsically disordered proteins such as α-synuclein and Aβ peptides with clustered distribution of charged residues in their primary sequences might bind to Aha1. To confirm and characterize the interaction between Aha1 and α-synuclein, in this study, ^1^H-^15^N-HSQC titration experiments were carried out (Figure 6). We then surprisingly found that both the N-terminal fragment M1-W27 and the C-terminal RLF (R^336^L^337^F^338^) motif of Aha1 contributed to the recognition of α-synuclein. This conclusion is supported by the previously reported data that the whole human Aha1 was required to keep denatured rhodanese in solution [25]. In our study, the important role of Aha1’s N-terminal fragment and C-terminal RLF motif in the recognition of α-synuclein was further confirmed by the ThT data and the TEM results, which demonstrated that Aha1 could interact with α-synuclein and consequently inhibit its aggregation process in vitro (Figure 6 and Figure 7).

In summary, by a combined use of multiple biochemical and biophysical techniques, we revealed that human Aha1 exhibited distinctive dynamics behavior and chaperone-like activity in solution. The unique structural and molecular dynamics features confer upon Aha1 both the co-chaperoning and chaperoning-like functions. In fact, Aha1 is not the only co-chaperone of Hsp90 that was found to show Hsp90-independent chaperone-like activity. Hsp90 co-chaperones including cis/trans PPIases FKBP51, FKBP52 and Cyp40, and non-tetratricopeptide repeat (TPR)-containing co-chaperone p23/Sba1 have also been reported to present chaperone activity in vitro [21,56,57,58]. It will be intriguing to unravel if and how the chaperoning functions of Aha1 and p23/Sba1 etc. are displayed in vivo. In particular, since Aha1 has been reported to drive the production of pathological tau aggregates by acting as Hsp90’s co-chaperone [59], which is in opposite to the inhibition effect of Aha1 on α-synuclein’s aggregation observed by us, extensive in vivo studies need to be conducted to precisely decipher the functional roles of Aha1 under different physiological and pathological conditions.

## 4. Materials and Methods

### 4.1. Plasmids

Full-length cDNA of Aha1 (Embank accession NM_012111) was synthesized by Generay Biotech Company, Limited (Shanghai, China). To create expression vectors designed to produce Aha1^28−162^, Aha1^204−335^, Aha1^28−335^ and Aha1^28−338^ proteins, the corresponding coding sequences for Aha1^28−162^, Aha1^204−335^, Aha1^28−335^ and Aha1^28−338^ were amplified by using polymerase chain reaction (PCR). The PCR products were digested with Nde1 and Xho1 and cloned into pET15b vector. All recombined plasmids were verified by gene sequencing. Expression vector for α-synuclein production was kindly provided by Prof. Conggang Li (Wuhan Institute of Physics and Mathematics, Chinese Academy of Sciences).

### 4.2. Protein Expression and Purification

Aha1 recombinant plasmids were transformed into BL21 (DE3) cells. Bacterial cells were grown in 10 mL of Luria Broth (LB) medium overnight at 37 °C and then transferred into 1 L LB medium containing Ampicillin (100 µg/mL) and grown at 37 °C. When the optical density (OD) value at 600 nm reached 0.6–0.8, the cells were induced by adding 0.5 mM isopropyl β-D-thiogalactoside (IPTG). The expression temperature was reduced to 15 °C and the cells were incubated for another 18 h. For the production of isotope-labeled (^15^N, ^13^C, Deuterium) Aha1, after growing in 10 mL of LB medium overnight at 37 °C, the bacterial cultures were centrifuged at 3000 rpm for 20 min, and the cell pellets were resuspended and transferred into 1 L M9 medium. When the OD value at 600 nm reached 0.4–0.6, the cells were induced by adding 0.5 mM IPTG. The expression temperature was reduced to 15 °C, and the cells were incubated for another 24 h to 40 h. The cells were then harvested by centrifugation and stored at −80 °C for future use. Frozen cells were resuspended in lysis buffer (20 mM Tris/HCl, 200 mM KCl, 20 mM imidazole, 5 mM β-mercaptoethanol, pH 7.5) and lysed by sonication on ice. The lysate was centrifuged at 11,000 rpm for 40 min, and the supernatant was loaded onto a Ni-NTA column. After washing the column by using 50 mL lysis buffer, thrombin protease was added to remove the His-tag of Aha1 protein samples. The eluted tag-free protein samples were then purified by size exclusion chromatography on a Superdex 75 column (GE Healthcare, Boston, MA, USA) with FPLC buffer (20 mM Tris/HCl, 150 mM KCl, 0.5 mM EDTA, 5 mM β-mercaptoethanol, pH 7.5). The produced protein samples were analyzed by SDS/PAGE (15% gel). Protein concentrations were determined by UV spectrophotometry.

α-synuclein was expressed in BL21 (DE3) cells by using a similar protocol as that applied in Aha1 production. When the OD value at 600 nm reached 0.6–0.8, the cells in LB medium were induced by adding 0.5 mM isopropyl β-D-thiogalactoside (IPTG) and incubated at 37 °C for another 6 h. After that, the cells were harvested by centrifugation and stored at −80 °C. Frozen cells were resuspended in lysis buffer (10 mM Tris/HCl, 1 mM EDTA, 1 mM PMSF, 1 mM DTT, pH 8.0) and lysed by sonication on ice. The lysate was then boiled for 30 min to denature the well-folded proteins, and the insoluble materials were removed by centrifugation. The supernatant from centrifugation was treated with streptomycin sulfate (10 mg/mL) at 4 °C for 30 min. After centrifugation, ammonium sulfate (360 mg/mL) was added into the supernatant, and the whole system was gently stirred at 4 °C for 30 min. The precipitates were resuspended using buffer A (20 mM Tris/HCl, pH 7.7) and further purified by ion exchange chromatography. The sample was loaded onto a HiTrap^TM^ Q HP column (GE Healthcare, Boston, MA, USA), and eluted with buffer A (20 mM Tris/HCl, pH 7.7)-buffer B (20 mM Tris/HCl, 1 M NaCl, pH 7.7) mixtures with stepwisely increased salt concentration. α-synuclein was finally eluted at around 200 mM NaCl. The purified α-synuclein was dialyzed to an MES buffer (10 mM 2-(N-Morpholino)ethanesulfonic acid, 150 mM NaCl, pH 7.5) for experimental use.

Representative SDS-PAGE results for the preparations of the aforementioned protein samples are shown in Supplemental Figure S9.

### 4.3. NMR Spectroscopy

To determine the solution structure of Aha1^28−162^, 2D and 3D NMR spectra including ^1^H-^15^N-HSQC, ^1^H-^13^C-HSQC, ^15^N-IPAP-HSQC, HNCO, HN(CA)CO, HNCA, HNCACB, CBCA(CO)NH, HBHA(CO)NH, H(CC)(CO)NH, (H)CC(CO)NH, HCCH-TOCSY, ^15^N-NOESY-HSQC and ^13^C-NOESY-HSQC were recorded on Bruker 600 MHz NMR spectrometer (Bruker, Billercia, MA, USA) equipped with a TCI cryoprobe at 25 °C. ^15^N- or ^13^C-single-labeled, ^15^N, 70% ^2^H-double-labeled and ^15^N/^13^C-double-labeled Aha1^28−162^ samples were used in the aforementioned NMR data acquisition. Meanwhile, ^1^H-^15^N-HSQC, ^15^N-IPAP-HSQC, HNCO, HN(CA)CO, HNCA, HNCACB, CBCA(CO)NH, HBHA(CO)NH, H(CC)(CO)NH and ^15^N-NOESY-HSQC spectra were acquired on Bruker 600 MHz or 900 MHz NMR spectrometers equipped with TCI cryoprobe at 25 °C for solution structure determination of Aha1^28−335^. ^15^N, 50% ^2^H-double labeled Aha1^28−335^, ^15^N, 70% ^2^H-double labeled Aha1^28−335^ and ^15^N/^13^C, 50% ^2^H-triple labeled Aha1^28−335^ samples were used in the aforementioned NMR data acquisition. Aha1^28−162^ and Aha1^28−335^ protein samples prepared for solution structure determination were concentrated to 0.5-1.0 mM in a Tris buffer containing 20 mM Tris/HCl, 150 mM KCl, 0.5 mM EDTA, 5 mM β-mercaptoethanol, 10% or 100% D_2_O (pH 7.5). For ^15^N-IPAP-HSQC data acquisition, 20 mg/mL filamentous virus (pf1 phage, ASLA Biotech) and 15 mg/mL filamentous virus were added into Aha1^28−162^ sample and Aha1^28−335^ sample, respectively, and the final concentrations of Aha1^28−162^ and Aha1^28−335^ were both 0.5 mM. NMR data were processed through NMRPipe program [60] and analyzed with CARA [61]_ENREF_40.

### 4.4. Structure Calculation

NOE-derived distance restraints, hydrogen bond restraints, dihedral angle restraints and RDC restraints were generated and applied in the structure calculations of Aha1^28−162^ and Aha1^28−335^ using XPLOR-NIH version 2.34. NOE-derived distance restraints were generated by the analysis of ^15^N-NOESY-HSQC and ^13^C-NOESY-HSQC spectra. Hydrogen bond restraints were defined according to the secondary structure element information derived from chemical shift index data and distinctive inter-strand NOEs. Backbone dihedral angle restraints (φ/ψ) were generated by TALOS+, using the chemical shifts of C, CA, CB, N and HN of the corresponding residues [62]_ENREF_41. The ^1^H-^15^N amide backbone RDCs were measured by the analysis of ^15^N-IPAP-HSQC spectra recorded under isotropic and partially aligned conditions [63,64,65]. The NOE-derived distance restraints, hydrogen bond restraints, dihedral angle restraints and RDC restraints were then inputted to calculate the solution structures of Aha1^28−162^ and Aha1^28−335^ through a simulated annealing algorithm. The restraints used for structure determination of Aha1^204−335^ (PDB 1X53) were directly transferred and applied in the structure calculation of Aha1^28−335^. Finally, 20 structures with the lowest energy from the ensemble of 100 calculated structures were selected to represent the solution structures of Aha1^28−162^ and Aha1^28−335^. The structure statistics were evaluated by the PROCHECK program [66]_ENREF_42_ENREF_33. The atomic coordinates of the determined solution structures of Aha1^28−162^ and Aha1^28−335^ have been deposited in Protein Data Bank (PDB), and the ID codes are 7DMD and 7DME.

### 4.5. NMR Relaxation Experiments

Rates for ^15^N longitudinal R_1_ and transverse R_2_ relaxation and magnitudes of the heteronuclear NOE enhancements were recorded on ^15^N, 50% ^2^H-double labeled Aha1^28−335^, Aha1^28−162^ and Aha1^204−335^. R_1_ and R_2_ relaxation rates were determined by using T_1_ and the T_2_ experiments, respectively. The data were collected on Varian 800 MHz NMR spectrometer at 25 °C with a protein concentration of 0.5 mM. All ^15^N-T_1_ and T_2_ relaxation experiments were carried out with a 3 s recycle delay between scans. For Aha1^28−335^, relaxation delays were set to 10, 20, 60, 100, 160, 320, 640, 1280, 1800 and 2560 ms for the T_1_ experiments and 10, 30, 50, 70, 90, 110, 130 and 150 ms for the T_2_ experiments. For Aha1^28−162^ and Aha1^204−335^, relaxation delays were set to 10, 40, 80, 160, 320, 640, 1280 and 2560 ms for the T_1_ experiments and 10, 30, 70, 90, 110, 150, 190 and 210 ms for the T_2_ experiments. R_1_ and R_2_ values were derived by fitting data acquired with different relaxation delays to a single-exponential decay function, and error values were assessed from Monte Carlo simulations. Heteronuclear NOE enhancements (XNOEs) were determined by calculating the ratios of the peak heights measured with and without proton saturation lasting for 2.5 s. Duplicate spectra were used to estimate experimental errors.

### 4.6. Dynamics Behavior Analysis

The internal motions of N-H bonds of Aha1^28−335^, Aha1^28−162^ and Aha1^204−335^ were elucidated by using the spectral density mapping approach [34,35,36,37,38]. The reduced spectral density values were calculated as follows:

(1) $$\sigma =\left(R_{1}\left(NOE-1\right)\gamma_{N}\right)/\gamma_{H}$$

(2) $$J\left(\omega_{N}\right)=\left(4R_{1}-5\sigma \right)/\left(3d^{2}+4c^{2}\right)$$

(3) $$J\left(0\right)=\left(6R_{2}-3R_{1}-2.72\sigma \right)/\left(3d^{2}+4c^{2}\right)$$

(4) $$J\left(0.87\omega_{H}\right)=4\sigma /5d^{2}$$

where *d* = *µ_0_hγ*_N_/*γ*_H_<*r*_NH_^−3^>/(8*π*^2^); *c* = ω_N_Δ*σ*/3^1/2^; *μ*_0_ is the permeability of the free space; *h* is the Planck’s constant; *γ*_H_ and *γ*_N_ are the gyromagnetic ratios of ^1^H and ^15^N, respectively; *r*_NH_ is the N-H bond length, ω_H_ and ω_N_ are the Lamor frequencies of ^1^H and ^15^N, respectively; and Δ*σ* is the chemical shift anisotropy for ^15^N with Δ*σ* = *σ*_∥_–*σ*_⊥_ = −160 ppm.

### 4.7. NMR Titration Experiments

^1^H-^15^N-HSQC NMR experiments were performed on Bruker 600 MHz or Varian 800 MHz NMR spectrometer at 25 °C. ^1^H-^15^N HSQC spectra of ^15^N-labeled Aha1, ^15^N-labeled Aha1^28−335^ and ^15^N-labeled Aha1^28−338^ were collected with or without the addition of 10-fold molar excess of unlabeled α-synuclein (1 mM). ^15^N-labeled Aha1, ^15^N-labeled Aha1^28−335^ and ^15^N-labeled Aha1^28−338^ were dissolved in a MES buffer containing 10 mM 2-(N-Morpholino)ethanesulfonic acid, 150 mM NaCl and 10% D_2_O (pH 7.5). In addition, to test whether Aha1’s N-terminal domain and C-terminal domain interacts with each other, ^1^H-^15^N HSQC spectra of ^15^N-labeled Aha1^28−162^/^15^N-labeled Aha1^204−335^ were recorded with or without the addition of equal molar of unlabeled Aha1^204−335^/unlabeled Aha1^28−162^. ^1^H-^15^N HSQC spectra of ^15^N, 50% ^2^H-double labeled Aha1^28−335^, Aha1^28−162^ and Aha1^204−335^ were collected with a protein concentration of 0.5 mM. The samples were prepared using a Tris buffer containing 20 mM Tris/HCl, 150 mM KCl, 0.5 mM EDTA, 5 mM β-mercaptoethanol and 10% D_2_O (pH 7.5). The spectra were processed by using NMRPipe [60] and analyzed with Sparky [67].

### 4.8. Thermal Shift Assay

The thermal shift assay was performed on a fluorescence quantitative PCR system (VII A7TM, ABI, CA, USA). Each reaction system contains 5 × SYPRO Orange, 15 µM protein samples dissolved in 20 µL of Tris buffer (20 mM Tris/HCl, 150 mM KCl, 0.5 mM EDTA, 5 mM β-mercaptoethanol, pH 7.5). The samples were heated from 25 °C to 99 °C at a rate of 0.05 °C/s, and the fluorescence intensities of the systems were monitored during the heating process. All of the thermal shift experiments were repeated three times, and the transition curves were processed using the Boltzmann equation.

### 4.9. In Vitro α-Synuclein Fibril Formation Assay

Seed fibrils were produced by following the procedure previously reported [68]. α-synuclein monomer at a concentration of 5 mg/mL was incubated at 37 °C in 1.5 mL microcentrifuge tube, with shaking for 7 days at 1000 rpm. In the case of seeded incubation, 1% (*v*/*v*) of α-synuclein fibrils were added to the 14 μM α-synuclein monomer solution, with the absence or presence of the 1.4 μM Aha1 incubated at 37 °C for three days (with shaking). Thioflavin T (ThT) and Electron Microscopy were applied to identify α-synuclein morphologies.

### 4.10. Thioflavin T Fluorescence Assay (ThT)

Fifty microliter protein samples and 12.5 μL ThT solution (25 μM) were incubated at 37 °C for 15 min, and the samples were then added to a 96-well, black plate (J09602, Jing An biological, China). Fluorescence was read at 450 nm excitation and 485 nm emission in a full-wavelength microplate reader (FlexStation3, Molecular Devices, LLC., San Jose, CA, United States). All conditions were performed at least in duplicate.

### 4.11. Electron Microscopy Experiments

Ten microliters of protein samples were absorbed onto square mesh copper grids (BZ11023a), incubated for 5 min and washed with 1% (*w*/*v*) uranyl acetate and then negatively stained with 1% uranyl acetate for 1min. Grids were viewed using a Talos L120C Electron Microscopy. At least three independent experiments were carried out for each sample.

## Figures and Tables

**Table 1:** Structural statistics for the calculated solution structures of Aha1^28−162^ and Aha1^28−335^.

	Aha1^28−162^		Aha1^28−335^
NOE Distance Restraints			
Total NOE	2210		6180
Intra-residual	728		1449
Sequential (|i-j| =1)	467		1375
Medium range (2≤ |i-j| ≤4)	425		1061
Long range (|i-j| >4)	590		2295
**Hydrogen Bonds**	143		136
**RDCs**	129		248
**Dihedral Angle Restraints**			
Total	238		452
Φ	119		226
Ψ	119		226
**Ramachandran Statistics**			
Most favored region (%)	81.9		81.5
Additionally allowed region (%)	14.1		16.5
Generously allowed region (%)	3.7		1.8
Disallowed region (%)	0.3		0.2
**RMSD from Mean Structure (Å)**	S33-T160	S33-T160	C207-F331
Backbone atoms	0.62 ± 0.11	0.67 ± 0.14	0.16 ± 0.04
Heavy atoms.	1.41 ± 0.23	1.55 ± 0.24	0.78 ± 0.06

NOE—Nuclear Overhauser Effect; RDC—Residual Dipolar Coupling; RMSD—Root Mean Square Deviation.

## Data Availability

The atomic coordinates of the determined solution structures of Aha1^28−162^ and Aha1^28−335^ have been deposited in Protein Data Bank (PDB), and the ID codes are 7DMD and 7DME.

## Supplementary Materials

Figure S1: 15N relaxation rates (R1, R2) and XNOEs of Aha128−162; Figure S2: 15N relaxation rates (R1, R2) and XNOEs of Aha1204−335; Figure S3: Superposition of 1H-15N-HSQC spectra of Aha128−335 and Aha128−162 or Aha1204−335; Figure S4: Structural ensemble of 20 best structures of human Aha128−335; Figure S5: The two domains of human Aha1 do not interact with each other in solution; Figure S6: 15N relaxation rates (R1, R2) and XNOEs of Aha128−335; Figure S7: Chemical shift changes for (a) Aha11−338 vs. Aha128−338 and (b) Aha11−338 vs. Aha128−335; Figure S8: Superposition of 1H-15N-HSQC spectra recorded on Aha11−338 and Aha128−335; Figure S9: Representative SDS-PAGE gels for the preparations of protein samples used in the study.
