# Supplementary material for: Aha1 Exhibits Distinctive Dynamics Behavior and Chaperone-Like Activity
Source: Molecules. 2021 Mar 30;26(7):1943. doi: 10.3390/molecules26071943 (PMC8037086; doi:10.3390/molecules26071943)
Supplement: Supplementary file 1 [file molecules-26-01943-s001.pdf]

Supporting information

Figure S1

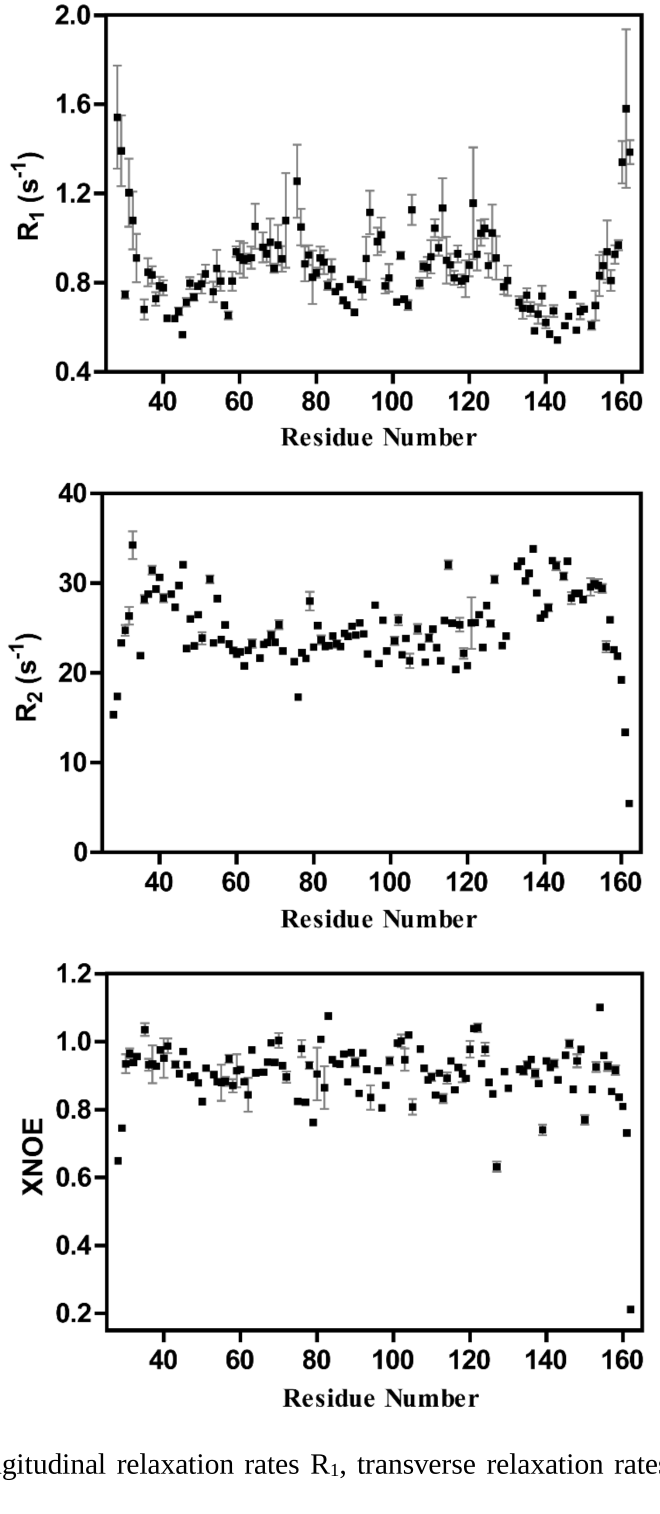

Figure S1. ^{15}N longitudinal relaxation rates R_1, transverse relaxation rates R_2 and $\{^1H\}$-^{15}N heteronuclear steady NOEs of the N-terminal domain of human Aha1 (Aha1$^{28\text{-}162}$) in its free state.

Figure S2

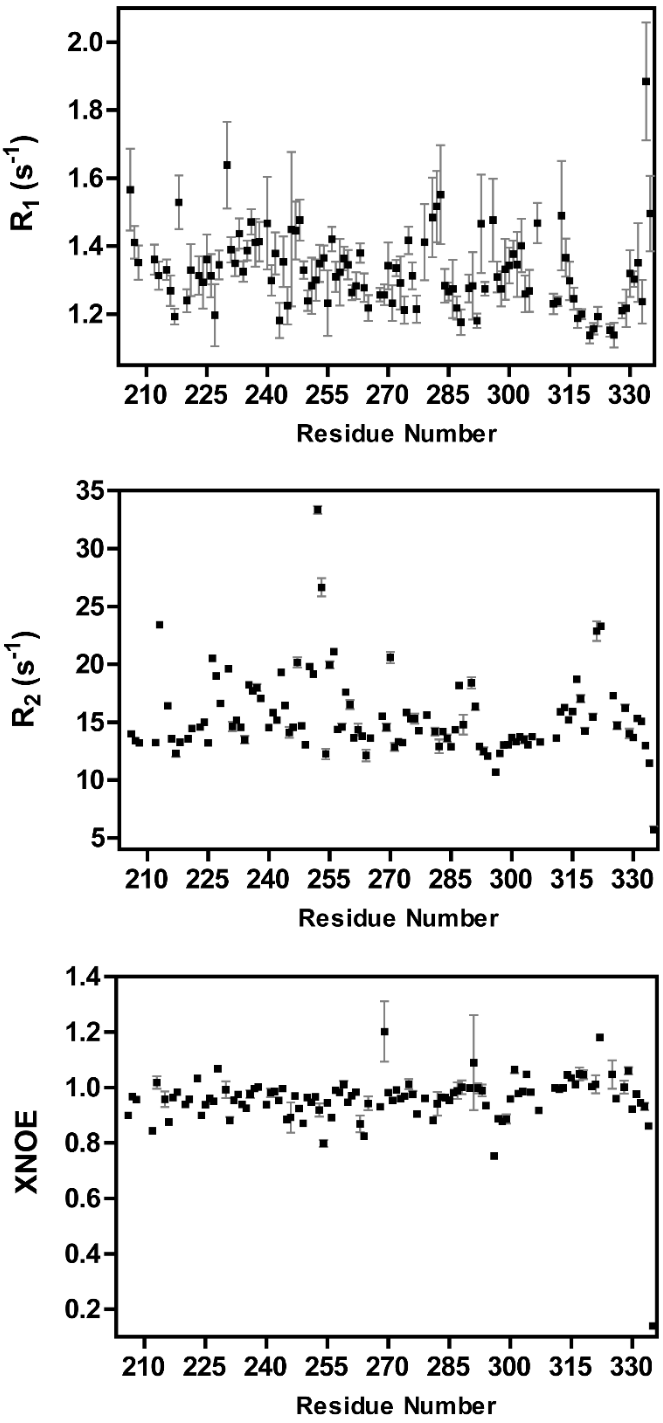

Figure S2. ^{15}N longitudinal relaxation rates R_1, transverse relaxation rates R_2 and $\{^1H\}$-^{15}N heteronuclear steady NOEs of the C-terminal domain of human Aha1 (Aha1$^{204\text{-}335}$) in its free state.

Figure S3

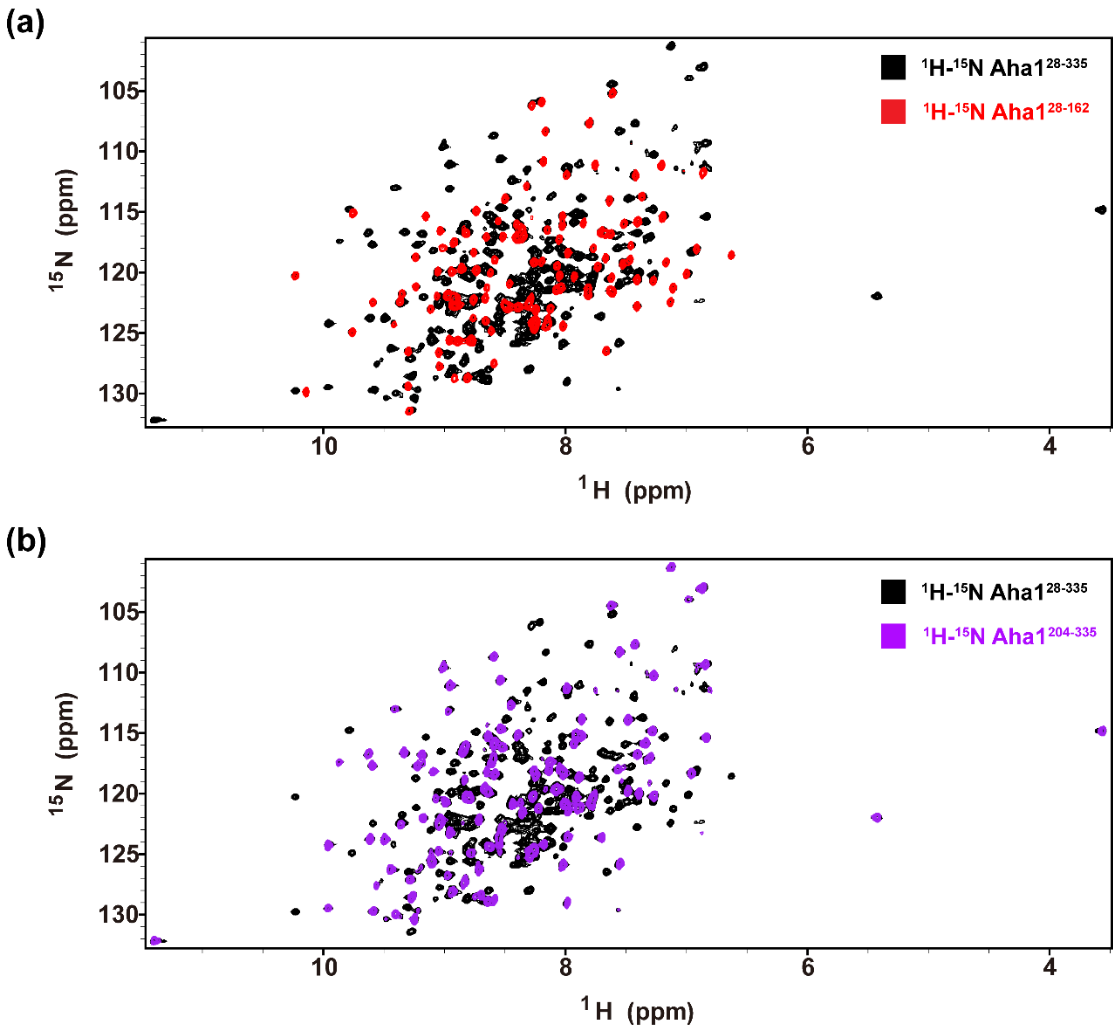

Figure S3. (a) Superposition of ^{1}H-^{15}N-HSQC spectra recorded on ^{15}N, 50% ^{2}H-double labeled Aha1$^{28\text{-}335}$ (colored in black) and ^{15}N, 50% ^{2}H-double labeled Aha1$^{28\text{-}162}$ (colored in red). (b) Superposition of ^{1}H-^{15}N-HSQC spectra recorded on ^{15}N, 50% ^{2}H-double labeled Aha1$^{28\text{-}335}$ (colored in black) and ^{15}N, 50% ^{2}H-double labeled Aha1$^{204\text{-}335}$ (colored in purple).

Figure S4

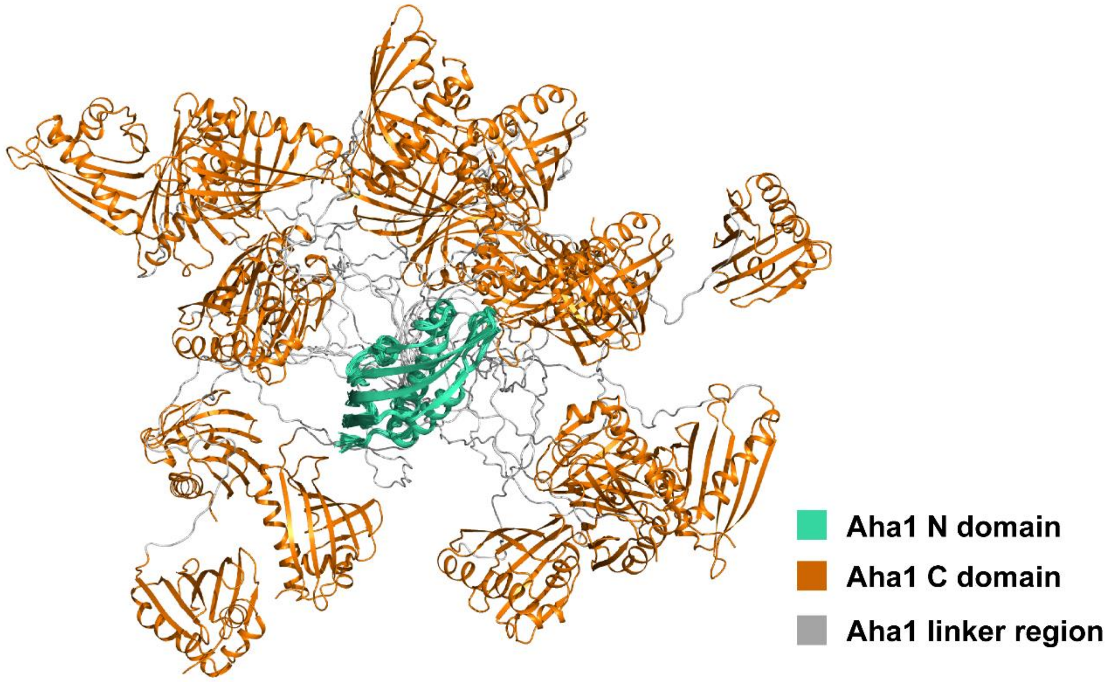

Figure S4. Structural ensemble of 20 best structures of human Aha1$^{28\text{-}335}$ (PDB code: 7DME). A jellyfish-like shape was formed when human Aha1's N-terminal domain served as an anchor in the superposition of Aha1$^{28\text{-}335}$'s solution structures.

Figure S5

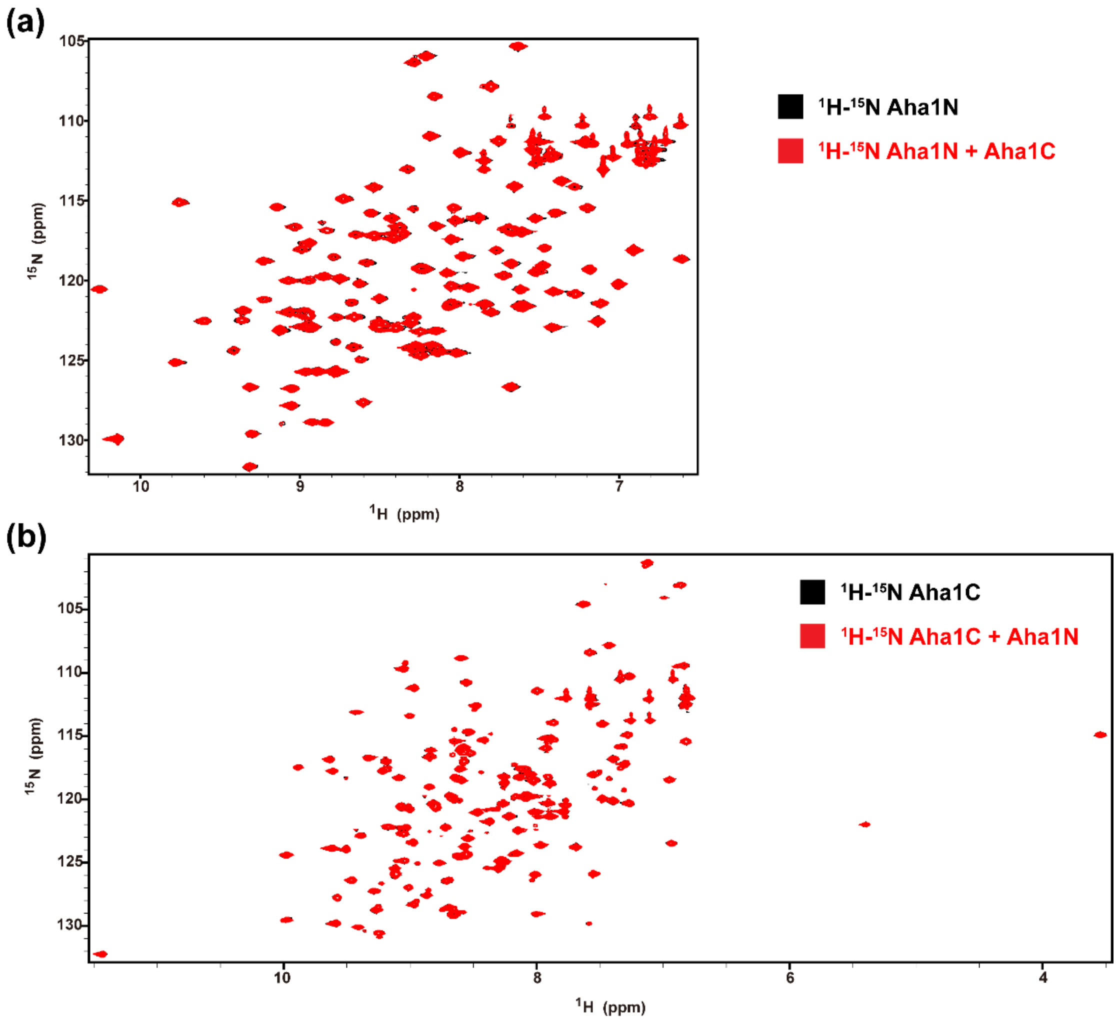

Figure S5. The two domains of human Aha1 do not interact with each other in solution. (a) Superposition of ^{1}H-^{15}N-HSQC spectra recorded on ^{15}N-labeled Aha1$^{28\text{-}162}$ (Aha1N) without (colored in black) or with (colored in red) the addition of an equal amount of unlabeled Aha1$^{204\text{-}335}$ (Aha1C). (b) Superposition of ^{1}H-^{15}N-HSQC spectra recorded on ^{15}N-labeled Aha1$^{204\text{-}335}$ (Aha1C) without (colored in black) or with (colored in red) the addition of an equal amount of unlabeled Aha1$^{28\text{-}162}$ (Aha1N).

Figure S6

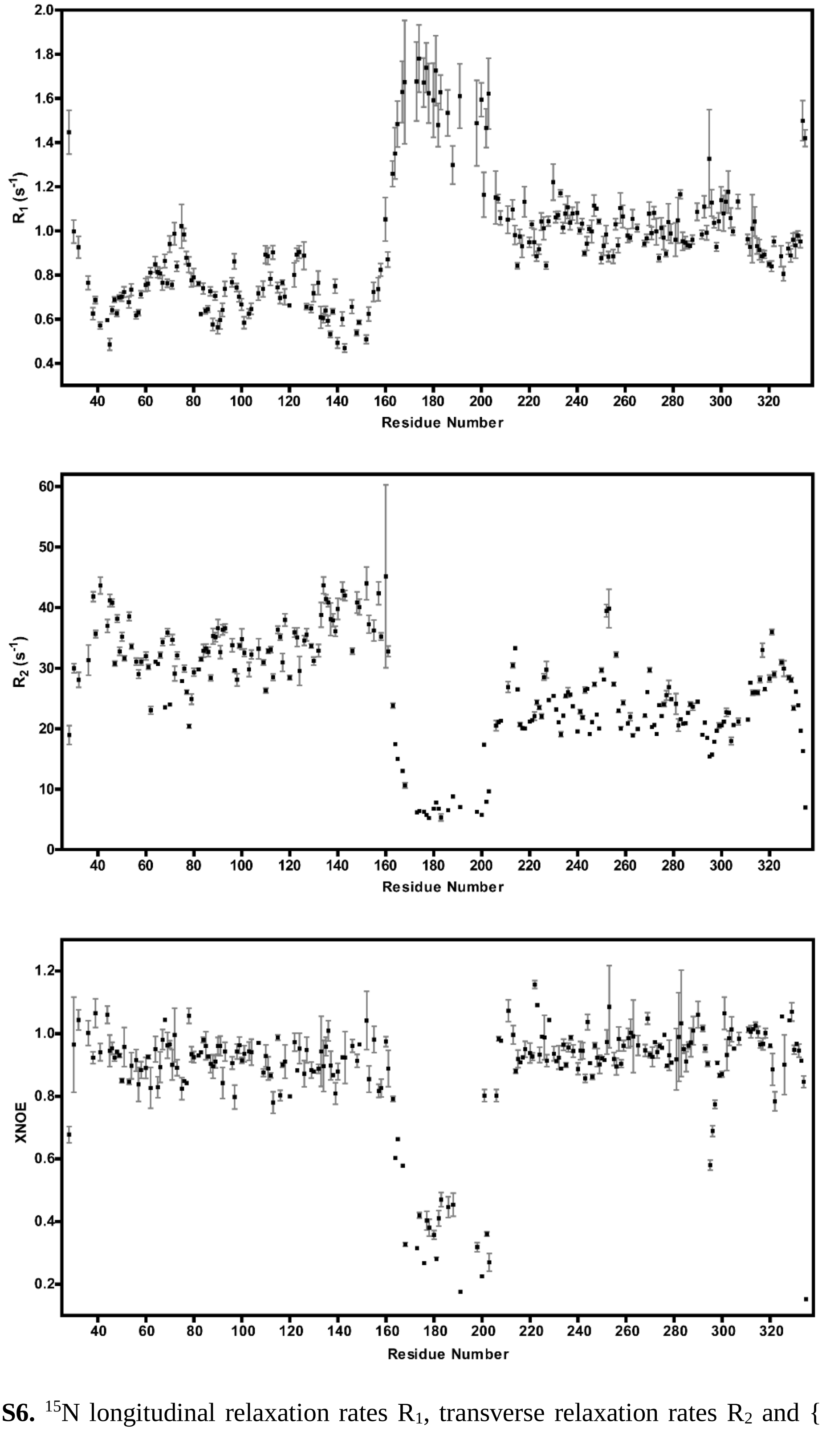

Figure S6. ^{15}N longitudinal relaxation rates R_1, transverse relaxation rates R_2 and {^{1}H}-^{15}N heteronuclear steady NOEs of Aha1$^{28\text{-}335}$ in its free state.

Figure S7

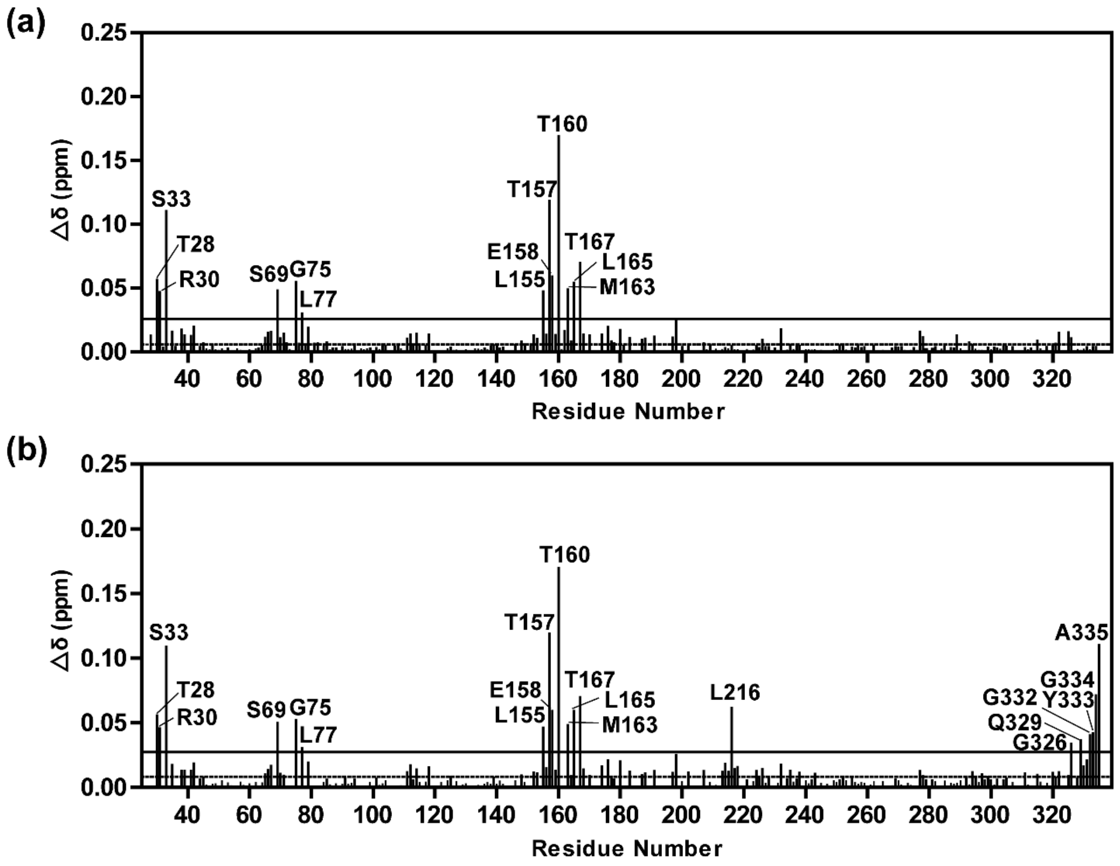

Figure S7. Chemical shift changes for (a) $Aha1^{1\text{-}338}$ *vs* $Aha1^{28\text{-}338}$ and (b) $Aha1^{1\text{-}338}$ *vs* $Aha1^{28\text{-}335}$ were calculated by using equation (1) listed below.

$$\Delta\delta = \sqrt{((\Delta\delta_N/5)^2 + \Delta\delta_H{}^2)/2} \quad (1)$$

Figure S8

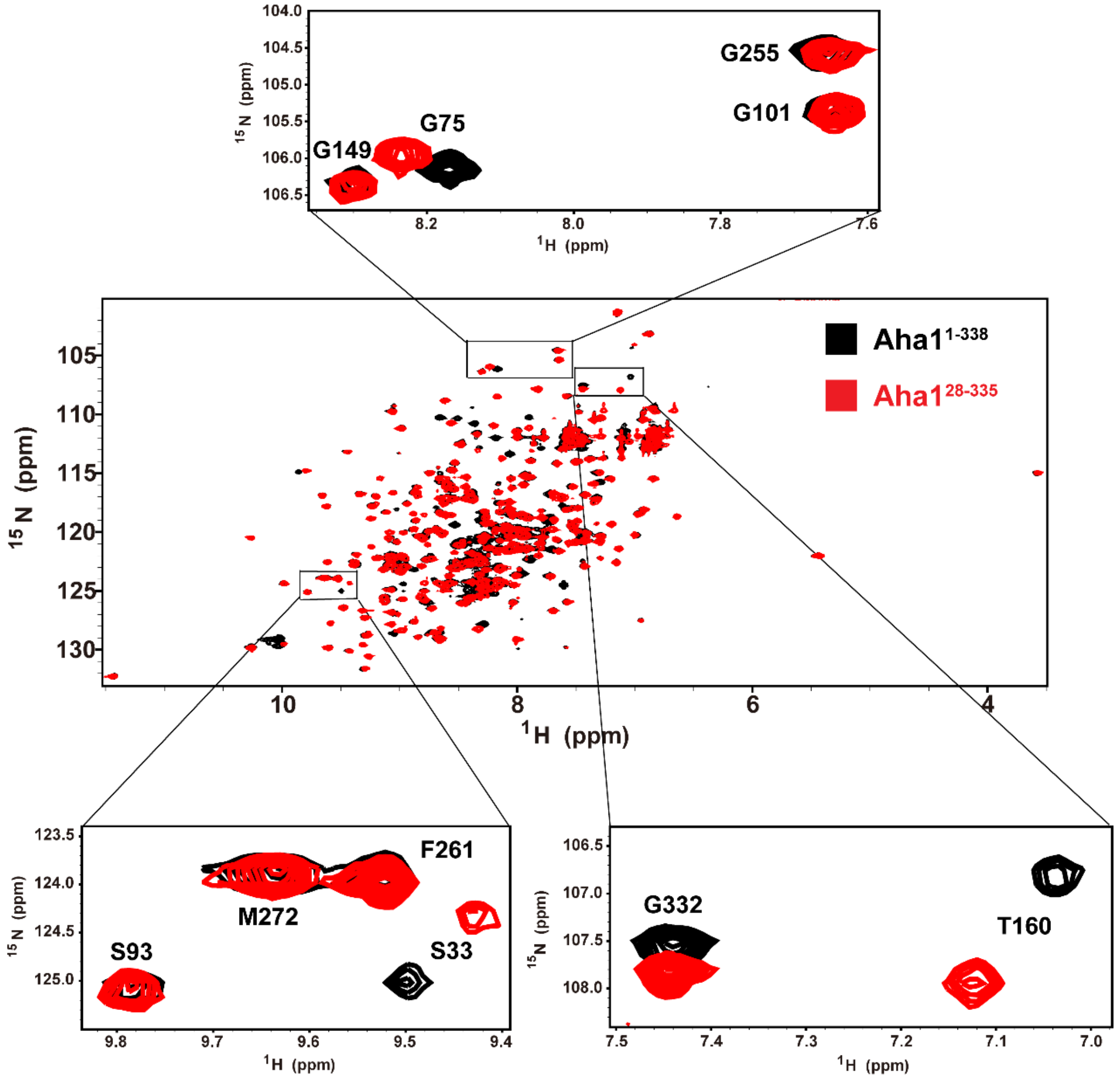

Figure S8. Superposition of ^{1}H-^{15}N-HSQC spectra recorded on ^{15}N-labeled Aha1$^{1-338}$ (colored in black) and ^{15}N-labeled Aha1$^{28-335}$ (colored in red). Selected ^{1}H-^{15}N-HSQC spectra regions are expanded to view representative residues which undergo resonance shifting upon the absence of Aha1's N-terminal fragment spanning M1-W27.

Figure S9

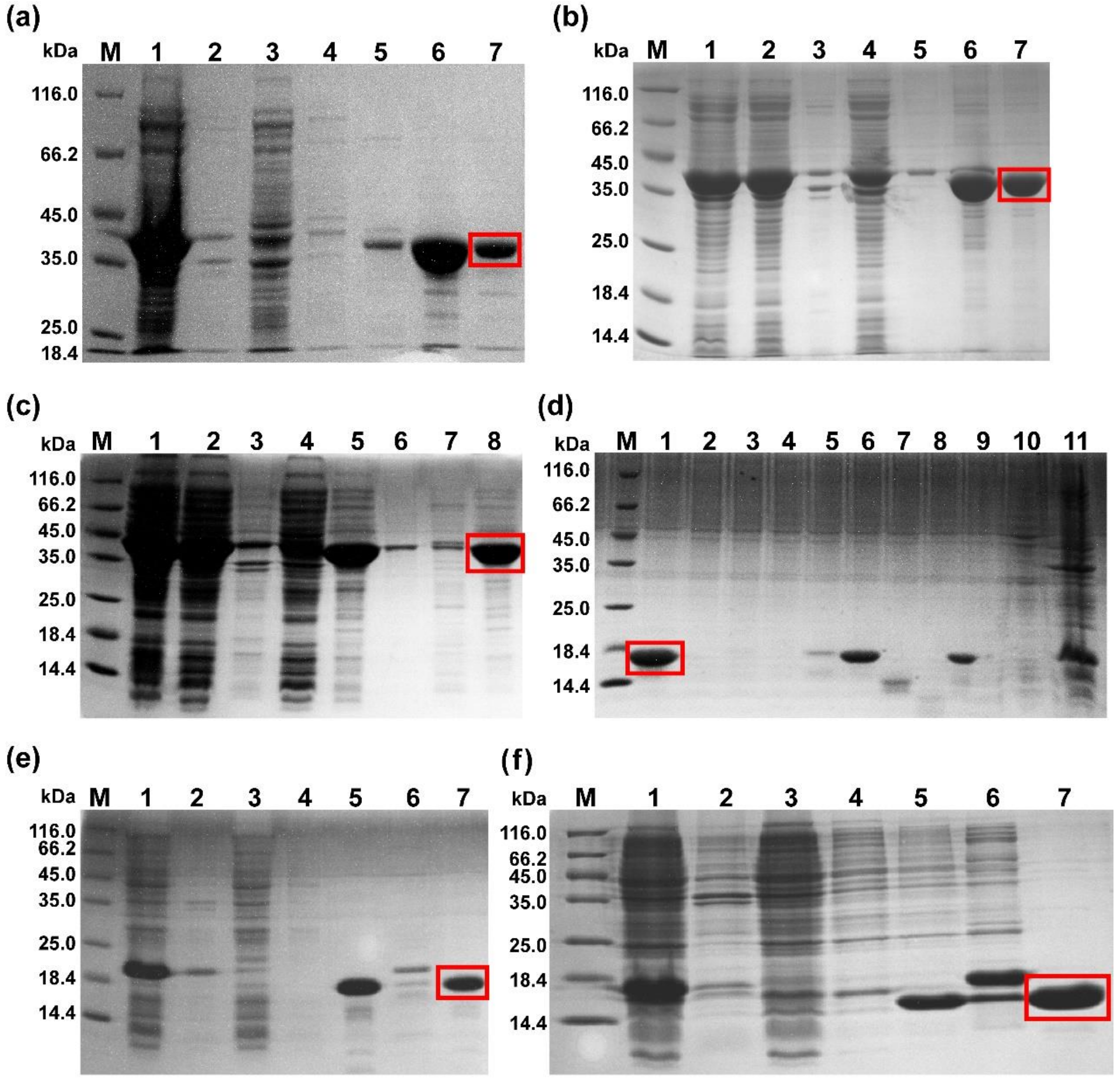

Figure S9. Representative SDS-PAGE gels for the preparations of protein samples used in the study. SDS-PAGE (15% gel) results for the purifications of (a) Aha1$^{28\text{-}338}$, (b) Aha1$^{28\text{-}335}$, (c) Aha1$^{1\text{-}338}$, (d) α-synuclein, (e) Aha1$^{28\text{-}162}$ and (f) Aha1$^{204\text{-}335}$. The protein molecular weight marker lane (26610, Thermo Scientific™) is labeled with M, and the gel staining results for the purified protein samples used in the experiments are highlighted with red box.
